# Ultrasound-guided biopsy of breast calcifications using a new image processing technique: initial experience

**DOI:** 10.1590/0100-3984.2017.0054

**Published:** 2018

**Authors:** Almir Galvão Vieira Bitencourt, Luciana Graziano, Camila Souza Guatelli, Maria Luiza Lima Albuquerque, Elvira Ferreira Marques

**Affiliations:** 1 MD, PhD, Radiologist at A.C.Camargo Cancer Center, São Paulo, SP, Brazil.; 2 MD, MSc, Radiologist at A.C.Camargo Cancer Center, São Paulo, SP, Brazil.; 3 MD, Radiologist at A.C.Camargo Cancer Center, São Paulo, SP, Brazil.

**Keywords:** Ultrasonography, mammary/methods, Breast diseases/diagnosis, Biopsy, needle/methods, Ultrassonografia mamária/métodos, Doenças mamárias/diagnóstico, Biópsia por agulha/métodos

## Abstract

The aim of this paper is to describe the use of a new ultrasound imaging processing technique to guide biopsies of suspicious breast calcifications. We used this technique in 13 patients with suspicious breast calcifications that could not be submitted to stereotactic biopsy. Suspicious calcifications were identified by ultrasound, and the biopsy was successfully performed in all cases. Although mammography continues to be the method of choice for the detection and characterization of microcalcifications, this new technique can be an alternative means of guiding biopsy procedures in selected patients who are not candidates for stereotactic biopsy.

## Abstract (pt)

O objetivo deste trabalho é descrever o uso de uma nova técnica de processamento de imagens na ultrassonografia para guiar biópsia de microcalcificações mamárias suspeitas. Utilizamos esta técnica em 13 pacientes que não puderam ser submetidos a biópsia por estereotaxia. As calcificações suspeitas foram identificadas na ultrassonografia e a biópsia foi realizada com sucesso em todos os casos. Apesar de a mamografia ser o método de escolha para detecção e caracterização de microcalcificações, esta técnica pode ser uma alternativa para guiar biópsia em pacientes selecionados em que não foi possível realizar biópsia estereotáxica.

## INTRODUCTION

Mammography is the method of choice to guide biopsies of suspicious breast calcifications^(1,2)^. However, despite the use of alternative approaches^(2,3)^, stereotactic biopsy is not technically achievable in all cases. Ultrasound has been proposed as an alternative to stereotactic biopsy in selected patients with suspicious breast calcifications^(4,5)^. However, conventional gray-scale examination limits the identification of calcifications, especially those located outside of a mass or duct, due to the lack of contrast with the normal breast parenchyma. Recent technical advances have improved the detection of calcifications by ultrasound^(6-8)^.

The aim of this paper is to describe the use of a new ultrasound imaging processing technique to identify and guide biopsies of suspicious breast calcifications detected on mammography.

## MATERIALS AND METHODS

From June 2014 through June 2016, 13 patients with suspicious grouped breast calcifications (< 2 cm in extent) that could not be submitted to stereotactic biopsy were submitted to ultrasound-guided biopsy after the identification of calcifications using a new imaging processing technique (MicroPure; Toshiba Medical Systems, Tokyo, Japan). MicroPure is an image processing function designed to enhance the visualization of microcalcifications by ultrasound. It combines nonlinear images and suppression techniques to highlight suspicious calcifications as white spots on a blue background. During ultrasound examination, gray-scale and MicroPure images are displayed side-by-side on the screen. All of the patients gave written informed consent prior to the procedure. Three radiologists with experience in breast imaging performed the ultrasound examinations and biopsies.

**Figure 1:**
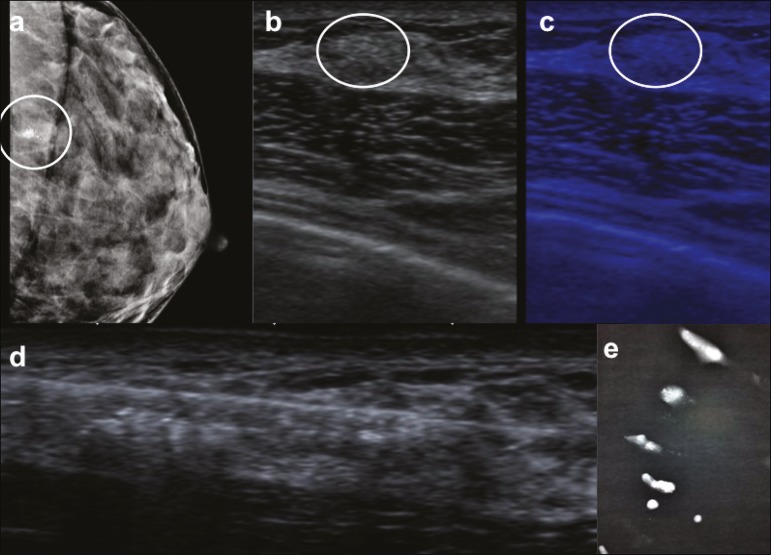
A 36-year-old asymptomatic woman at high risk for breast cancer. **a:** Mammography showing grouped amorphous calcifications in the left breast (circle), near the chest wall, which precluded the use of stereotactic biopsy. **b,c:** Ultrasound showing the calcifications (circle) on gray-scale imaging (**b**) and MicroPure imaging (**c**). **d:** Ultrasound-guided 12-gauge core needle biopsy, the pathological analysis of which was consistent with atypical ductal hyperplasia. **e:** X-ray image of the specimens, confirming the presence of calcifications.

Mammography images were analyzed to evaluate the distribution and topographic location of the calcifications. Ultrasound was then performed on an Aplio 500 system (Toshiba Medical Systems, Tokyo, Japan) with a high-frequency linear transducer. After identification of the suspicious calcifications on ultrasound, 12-gauge core needle biopsy was performed. Specimen X-ray images were obtained to confirm that the calcifications were present (Figure 1).

## RESULTS

In all 13 of the cases evaluated, stereotactic biopsy could not be performed, because of the location of the lesion in five cases (the lesion being near the skin in one, near the chest wall in one, and near breast implants in three); because of insufficient thickness of the compressed breast in three; and because of clinical conditions in five (one obese patient, one patient who refused to undergo the procedure, one patient with abdominal sarcoma, and two elderly patients with comorbidities that prevented them from assuming the prone position). Suspicious calcifications were identified using ultrasound, and the biopsy was successfully performed in all cases, without complications. The main technical difficulty was to identify suspicious calcifications after removal of the first sample, due to the presence of gas and edema/hematoma at the biopsy site. However, that did not impede the removal of calcifications in the additional samples. Chest X-ray revealed microcalcifications in all specimens. Table 1 summarizes the characteristics of the lesion, pathological results, and outcomes.

**Table 1:** Characteristics of the lesions, pathological results, and outcomes in patients submitted to ultrasound-guided biopsy of suspicious breast calcifications.

Age, years	Mammography finding	BI-RADS	Pathology	Outcome
30	Grouped amorphous calcifications	4	High-grade DCIS	Confirmed on surgical resection
35	Segmental coarse heterogeneous calcifications	5	High-grade DCIS	Patient in treatment for advanced abdominal sarcoma
36	Grouped amorphous calcifications	4	Atypical ductal hyperplasia	Upgrade on surgery to intermediate-grade DCIS
43	Mass with calcifications	4	Mucinous carcinoma	Confirmed on surgical resection
46	Grouped amorphous calcifications	4	High-grade DCIS	Confirmed on surgical resection
49	Mass with calcifications	4	Fibroadenoma	Stable on imaging follow-up for 20 months
51	Grouped amorphous calcifications	4	Fibroadenomatoid changes	Stable on imaging follow-up for 12 months
53	Segmental fine pleomorphic calcifications	4	High-grade DCIS	Confirmed on surgical resection
55	Grouped amorphous calcifications	4	Fibrocystic disease	Confirmed on surgical resection
56	Grouped amorphous calcifications	4	Atypical lobular hyperplasia	Confirmed on surgical resection
63	Grouped fine pleomorphic calcifications	4	Fibroadenomatoid changes	Stable on imaging follow-up for 24 months
75	Grouped fine pleomorphic calcifications	4	High-grade DCIS	Confirmed on surgical resection
76	Grouped fine pleomorphic calcifications	4	DCIS and IC-NST	Confirmed on surgical resection

DCIS, ductal carcinoma in situ; IC-NST, invasive carcinoma of no special type.

## DISCUSSION

The technique described here improves the capacity of ultrasound to detect calcifications and increases the confidence of radiologists in their ability to perform ultrasound-guided biopsy in selected cases, when there are technical challenges for stereotactic biopsy due to the lesion location or clinical characteristics of the patients. There have been few reports on the clinical utility of the MicroPure technique in locating and evaluating breast calcifications^(7,8)^. Park et al.^(6)^ recently used the MicroPure ultrasound function to guide biopsies of suspicious calcifications in nine patients. As in the present study, ultrasound biopsy was successfully performed in all patients. Further large-scale studies are needed in order to assess the potential contribution of this new technique and to compare its performance with that of stereotactic biopsy in the diagnosis of suspicious calcifications.

In conclusion, although mammography remains the gold standard for the histological evaluation of suspicious breast calcifications, ultrasound using innovative imaging processing techniques can be an alternative means of guiding biopsies, thus avoiding unnecessary surgery, in selected patients.
